# Flux Balance Analysis with Objective Function Defined by Proteomics Data—Metabolism of *Mycobacterium tuberculosis* Exposed to Mefloquine

**DOI:** 10.1371/journal.pone.0134014

**Published:** 2015-07-28

**Authors:** Daniel Montezano, Laura Meek, Rashmi Gupta, Luiz E. Bermudez, José C. M. Bermudez

**Affiliations:** 1 Laboratório de Pesquisa em Processamento Digital de Sinais (LPDS), Federal University of Santa Catarina (UFSC), Florianópolis, SC, Brazil; 2 College of Veterinary Medicine, Oregon State University, Corvallis, OR, United States of America; 3 College of Medicine, University of Central Florida, Orlando, FL, United States of America; Université de Nantes, FRANCE

## Abstract

We present a study of the metabolism of the *Mycobacterium tuberculosis* after exposure to antibiotics using proteomics data and flux balance analysis (FBA). The use of FBA to study prokaryotic organisms is well-established and allows insights into the metabolic pathways chosen by the organisms under different environmental conditions. To apply FBA a specific objective function must be selected that represents the metabolic goal of the organism. FBA estimates the metabolism of the cell by linear programming constrained by the stoichiometry of the reactions in an *in silico* metabolic model of the organism. It is assumed that the metabolism of the organism works towards the specified objective function. A common objective is the maximization of biomass. However, this goal is not suitable for situations when the bacterium is exposed to antibiotics, as the goal of organisms in these cases is survival and not necessarily optimal growth. In this paper we propose a new approach for defining the FBA objective function in studies when the bacterium is under stress. The function is defined based on protein expression data. The proposed methodology is applied to the case when the bacterium is exposed to the drug mefloquine, but can be easily extended to other organisms, conditions or drugs. We compare our method with an alternative method that uses experimental data for adjusting flux constraints. We perform comparisons in terms of essential enzymes and agreement using enzyme abundances. Results indicate that using proteomics data to define FBA objective functions yields less essential reactions with zero flux and lower error rates in prediction accuracy. With flux variability analysis we observe that overall variability due to alternate optima is reduced with the incorporation of proteomics data. We believe that incorporating proteomics data in the objective function used in FBA may help obtain metabolic flux representations that better support experimentally observed features.

## Introduction

Flux balance analysis (FBA) is a popular method for estimating metabolism of prokaryotic organisms under different environmental conditions such as hypoxia or nutrient starvation. The use of FBA requires the specification of an objective function representing the metabolic goal of the cell for each condition. Several objective functions have been proposed [1] and may be used in FBA as the assumed metabolic goal of the cell. Some objective functions result in linear optimization problems while others require the formulation of a quadratic optimization problem. A commonly used objective function for FBA analysis is the biomass synthesis equation, usually defined in the *in silico* metabolic model by a convenience equation accounting for the proportions of the metabolic precursors and macromolecules needed for cell growth (e.g. DNA, RNA, protein content, other small molecules, etc.). This is, for instance, the approach followed by the E-flux method [2]. The biomass function works well in a great variety of problems and several biologically validated insights have been obtained with this objective [3]. However, in some situations, the maximization of biomass yield may not represent the best biological goal of the cell. One such example is when the cell is exposed to an antibiotic. In these cases the goal of the organism is clearly not growth or replication, but rather survival, and therefore a different objective function should be used to study metabolism in such cases.

Proteomics data can be reliably obtained for a large number of proteins. Experiments are relatively ubiquitous nowadays, together with other experiments that generate large amounts of data such as DNA microarrays and metabolomics. By performing proteomics experiments with bacterial cultures growing under control and treatment conditions, the fold-change measurements obtained are representative of the relative change in protein content from one condition to the other. It is known that metabolic fluxes are, although not exclusively, dependent on the relative amounts of the corresponding enzymes that catalyze each reaction. Assuming that enzyme levels in prokaryotic organisms are closely related to their respective fluxes, we propose to construct the FBA objective function using this information. We conjecture that this new objective function is better suited to study the metabolism of the organism under exposure to an antibiotic than the more commonly assumed objective of optimal growth.

Several methods have tried incorporating different sources of data such as transcriptomics, proteomics, metabolomics and regulation into the FBA framework to improve the inferences made by the method and to broaden the spectrum of applications in which the method can be applied [2, 4, 5]. Although we do not claim that a proteomics-based objective function may be a substitute for situations where clearly the optimal growth assumption may be sound, we believe that for cases of antibiotic stress, this may be a better option for a metabolic objective. A method closely related to the one we propose here is the E-flux method described in [2], which was originally proposed in the context of transcriptomic data. Nevertheless, in [2] the authors suggest that the use of proteomics data may be of value and likely to improve FBA predictions. This method has been evaluated in comparison with several other methods in a recent review [5] of FBA techniques that propose transcriptomic data integration. Since it is known that there is a gap of information between RNA measurements and enzyme measurements, it is possible that by designing methods that are more specific to proteomics data we may improve prediction quality.

In this paper we present results of flux balance analysis for the *Mycobacterium tuberculosis* bacterium when exposed to mefloquine, a candidate anti-tuberculosis compound that has shown efficacy against the organism in previous studies [6]. We propose to use proteomics data in the definition of the objective function for FBA to study the *in silico* metabolic behavior of the bacterium when under stress. The results of the model are compared with enzyme essentiality data available in [7] and with the alternative E-flux method in terms of enzyme abundances.

The paper is a follows. In section Materials and Methods we describe the data resulting from the proteomics experiments, present some details of the FBA methodology, the procedure proposed to define the objective function from proteomics data and a description of the validation methodology. In section *Results and Discussion* we present and discuss the results and compare them with biological knowledge available in databases and in the literature. In section *Conclusion* we present the main advantages of using proteomics data to define objective functions in flux balance analysis and ideas for refinement and further development of the technique.

## Materials and Methods

### Tuberculosis and Mefloquine

Mycobacterial infection and more specifically tuberculosis, represents one of the major epidemiological challenges of the world. Much improvement in drug development can be obtained with a better understanding of the physiology and metabolism of the causing agents of the disease [8]. *Mycobacterium tuberculosis* is a highly adaptable bacterium that causes tuberculosis. Although several anti-tuberculosis compounds, both first- and second-line, are available to kill the bacterium, its ability to rapidly mutate requires new compounds to be actively researched [9]. It has been shown that mefloquine, an anti-malarial compound, is bactericidal against *Mycobacterium avium* and *Mycobacterium tuberculosis*, and no resistant mutants could be obtained from *in vitro* screening [6]. It is therefore possible that a new anti-tuberculosis compound may be derived from mefloquine as more research on its properties and mechanism of action, such as the present work, come to light.

### Proteomics Data

Bacterial cultures were essentially performed as described in [10, 11]. **Bacteria**: *Mycobacterium tuberculosis* H37Rv was used in the studies (American Type Culture Collection, Manassas, Virginia 20110, USA). Bacteria were cultured in Middlebrook 7H10 agar supplemented with albumin, dextrose and catalase. **Proteomic Analysis**: *M. tuberculosis* (100 ml in 500 ml flask) with a cell density of 1 × 10^8^ cells/ml was grown for 7 days in Middlebrook 7H9 broth supplemented with ADC. DMSO (carrier/solvent) or 8 mcg/ml of mefloquine (Sigma Co, St Louis, MO) dissolved in DMSO. Cultures were then harvested by centrifugation at 3,000 r.p.m. for 10 minutes at time intervals of 6h, 2 and 4 days. Cell pellets were washed 3 times in saline/tween (0.8% w/v NaCl/ 0.05 v/v Tween 80) solution. The pellets were resuspended in bacterial cell lysis buffer (Sigma) containing 80 mcl of protease inhibitor cocktail. Bacterial cells were lysed by bead beating 3x (speed of 8 per 2 min) using a mini BeadBeater and kept on ice for an additional number of minutes between sessions.

Samples were then centrifuged at 15,000 rpm at 4°C for 20 min, and the soluble fraction was transferred to a new tube and stored at −20°C. Soluble proteins samples were run on a 12% NuPage Bis Tris gel and processed through in-gel trypsin digestion (Promega MAX surfactant trypsin enhancer) for mass spectrometry. Mass spectrometry was performed on a termo Scientific LTQ-FT-MS Ultra System (Kalamazoo, MI) and the analysis was made using Scaffold 4 software (Proteome Software, Corvallis, OR). In Scaffold, the minimum characteristics for protein identification confidence were set as 99% for *protein threshold*, *minimum number of peptides* equal to 2 and *peptide threshold* of 95%. Quantitative value (*normalized total spectra*) were used for subsequent analysis. Scaffold viewer is freely available for download from the Proteome Software website [12]. Proteomic analysis was repeated two times and mean values of each experimental condition and time point were used for subsequent FBA calculations. Data is available as supporting information S1 Dataset. Table 1 shows these measurements for a sample of the most highly expressed proteins at each time point. Proteins are identified by their respective locus tag for the H37Rv strain [13]. In this paper, the locus tag for the H37Rv strain for each protein is followed by the respective UniProt entry acession number in parentheses the first time the protein is mentioned. In the following section we briefly review the FBA technique and explain in detail the proposed method for defining a proteomics-based objective function.

**Table 1:** Fold change for top over expressed proteins per time point.

Time point	Locus tag	Uniprot entry	Fold-change
6 hrs	*Rv2987c*	I6YAT3	55
6 hrs	*Rv1311*	I6Y678	30
6 hrs	*Rv1437*	I6X185	56
6 hrs	*Rv1311*	I6Y678	7.1
Day 2	*Rv2334*	I6Y910	37
Day 2	*Rv2868c*	I6YEL0	30
Day 2	*Rv0155*	P96832	44
Day 2	*Rv1001*	I6X008	29
Day 4	*Rv2831*	I6YEH6	37
Day 4	*Rv0382c*	I6Y3M7	29
Day 4	*Rv0500*	I6Y7Z2	34
Day 4	*Rv0753c*	053816	34

This table shows proteins with largest fold-change values between mefloquine and control experimental conditions.

### Flux Balance Analysis

The core procedure of the flux balance technique has been thoroughly described elsewhere [14, 15]. It is a linear optimization method for studying metabolism based solely on stoichiometric knowledge of the organism’s network of biochemical reactions without relying on knowledge of enzyme kinetics. By constructing an *in silico* model of the metabolic network and defining a suitable metabolic objective function, FBA then identifies an optimal metabolic phenotype as a vector of fluxes for each reaction present in the model. A large genome-scale *in silico* network of the *Mycobacterium tuberculosis* has already been constructed and is presented in [16]. The *in silico* reconstruction of this GSMN (*Genome-Scale Metabolic Network*) comprises 856 metabolic reactions involving metabolites with 726 specific enzymes catalyzing associated reactions. The model is available in the SBML (Systems Biology Markup Language) and CSV formats in S1 File. The SBML format can be used in several standalone packages with parsers for the format (such as JyMet explained below), while the CSV format is usually more flexible for inclusion in text processing pipelines and simple scripts written in languages yet without specific SBML parsers.

Several methods have already been presented that incorporate experimental data onto FBA methods to achieve metabolic predictions that better characterize the different environmental and experimental conditions [4, 17]. Data is usually incorporated in the form of hard or soft constraints, and when both transcriptomics and proteomics data are used, it is to solve for inconsistencies between measurements of transcriptional and translational activities [18]. To the best of our knowledge, experimental data has not been used in the specification of the objective function. In this work we propose the use of proteomics to determine the FBA objective function. In this study we are attempting to estimate the complete metabolic state of the bacterial cell using an *in silico* model. Although it is clear that quantitative enzyme information by itself (without using e.g. metabolomics, signalling proteins, pathway knowledge, RNA expression, etc.), when used in a *in silico* model to improve results of an optimization procedure cannot produce a complete picture of the metabolism under drug exposure, it is our conjecture that it can produce a better characterization of the metabolism in situations where the assumption of an objective function for optimal growth (maximization of biomass) can not be made.

Simulation scripts for all the FBA calculations used in this paper were written in the R programming language and are available in the supplementary data file S1 File. For solving the flux balance analysis optimization problems we used a freely available wrapper on the GLPK (*GNU Linear Programming Kit*) called SurreyFBA. The program is freeware and available for download from (http://sysbio3.fhms.surrey.ac.uk/). Its main features have been presented in [19]. The program can be used as a CLI (command-line interface) program, which is more suitable for inclusion in the R scripts used in our simulations, but also as a GUI (graphical user interface) using the JyMet frontend, that is included with SurreyFBA. The SurreyFBA program accepts *in silico* metabolic models in both SBML format and simpler CSV text files, and both these models are also included as supplementary information S1 File. Alternatively, several other packages are also available for performing similar calculations [20].

### Defining the Objective Function from Proteomics Data

In this section we describe our proposed objective function. We explain how proteomics data can be used to determine an objective function for FBA in cases where the organism is under drug-induced stress, and thus optimal growth should not be assumed as the metabolic goal of the cell. We propose to define the objective to be a linear combination of fluxes as in [2, 21]. However, the weights associated to the different fluxes are determined in a different manner. The objective function is the linear combination

$$\begin{array}{c} f\left(v\right)=\sum_{k=1}^{N}c_{k}v_{k}=c^{T}v \end{array}$$ (1)

where ***v*** = [*v*_1_, *v*_2_, …, *v*_*N*_] is the flux vector, and ***c*** is the vector of coefficients of the linear combination. The dimension *N* of both vectors is equal to the number of reactions (i.e. metabolic fluxes) in the model. In the case of biomass maximization, vector ***c*** is an all-zero vector except for a one (1.0) in the position corresponding to the biomass reaction:

$$\begin{array}{c} f_{bio}\left(v\right)=c^{T}v=\left[1,0,0,\ldots ,0\right][\begin{array}{c} v_{bio}\\ v_{1}\\ v_{2}\\ \vdots \end{array}] \end{array}$$ (2)

where *v*_*bio*_ is the corresponding flux for biomass in the model. In our method we propose to use the same linear combination of fluxes for the objective function, but instead of maximizing the biomass flux, we calculate the coefficients *c*_*k*_ from proteomics data.

We first define a vector ***p*** containing the quantitative values of protein levels obtained with the proteomics experiment (cf. Materials and Methods) for one experimental condition and time point. Vector ***p*** is of length *K* and given by:

$$\begin{array}{c} p=[\begin{array}{c} p_{1}\\ p_{2}\\ \vdots \\ p_{K} \end{array}] \end{array}$$ (3)

where each element *p*_*k*_ corresponds to a value representing the level of protein *k* in the sample. While proteomics experiments are very precise and reliable (as opposed to microarray experiments, where the signal-to-noise ratio is much lower), the number *K* of identified proteins is only a subset of the whole proteome of the cell, a difficulty stemming from two facts; (1) the method performs identification by comparison of peptide fragments against a database, so that only previously identified proteins are accounted for and (2) not all proteins may be susceptible to the cleavage procedure.

Moreover, since some proteins may not appear in a specific sample (e.g. protein A may be present in sample *6h control* but not in sample *day 1 mefloquine*), we set the levels of all proteins absent from a sample to zero. It is also important to note that while the proteome experiment quantifies both enzymes and signaling proteins, vector ***p*** contains only the subset of proteins with enzymatic activity and that are present in the *in silico* model. Although this leaves a number of important regulatory proteins not accounted for, we propose to include regulatory information in a subsequent analysis.

As a second step, vector ***p*** is normalized by its maximum value to yield a vector of relative protein levels $\tilde{\text{p}}$:

$$\begin{array}{c} \tilde{p}=p/\max\left\{p\right\} \end{array}$$ (4)

where $\tilde{\text{p}}$ is also of dimension *K*. This normalized vector will be used in the expressions from the GSMN-TB model that combine enzyme actions, as we explain shortly. It is important to note that each value ${\tilde{p}}_{k}$ is associated with one enzyme.

Following the normalization procedure we identify which reactions in the metabolic model are catalyzed by the enzymes represented in $\tilde{\text{p}}$. The values of the elements ${\tilde{p}}_{k}$ of $\tilde{\text{p}}$ are then used to determine the values of the coefficients *c*_*k*_ for our proposed objective. In the simplest case where each enzyme catalyzes one reaction, we set the corresponding coefficient *c*_*k*_ of each reaction to the respective value ${\tilde{p}}_{k}$ of the catalyzing enzyme. For all the other fluxes, i.e. those not present in $\tilde{\text{p}}$, the respective coefficients are set to zero since these represent proteins *not* observed in the proteomics experiment.

More complex situations arise when one reaction is catalyzed not by a single enzyme, but by the combined action of a set of enzymes. In such situations, the determination of the coefficients *c*_*k*_ of the objective function depends on the relationship between enzymes and metabolic reactions. Therefore, it is important to understand how enzymes and reactions are related in the metabolic model, and how this information is used in the definition of the proposed objective function. In the GSMN-TB model, metabolic reactions that are catalyzed by a set of enzymes are associated with a boolean expression that combines the action of all enzymes needed for catalysis of the reaction. These expressions were manually curated by the authors of the GSMN-TB model and more details and references can be found in [16]. As an example, let us check model reaction R022 (myo-inositol synthesis). For this reaction the catalytic proteins are combined in the following boolean expression:

$$\begin{array}{c} \text{Rv0046c}\vee (\text{Rv2612c}\wedge \text{Rv1822}) \end{array}$$ (5)

where the operators ∨ and ∧ represent the boolean operators OR and AND respectively. The proteins in this expression are the two transferases *Rv2612c* (IGY178), *Rv1822* (I6X2EA) and the synthase *Rv0046c* (I6X8D3), all involved in inositol synthesis [22].

In these cases, to obtain the coefficients for the fluxes that will be used in the objective function, it is first necessary to substitute the proteomics data in $\tilde{\text{p}}$ into the corresponding boolean expressions (similar to Eq (5)). The mathematical operation for the boolean OR results in the maximum of the terms, while the AND operation results in the minimum of the two terms, in a similar fashion as done in [2]. The resulting values from the boolean expressions are used as the final values for the elements in **c** of coefficients for the objective function. We note that vector **c** will have a number of non-zero elements that is usually different from *K*, unless we have the very specific situation mentioned before where each reaction is catalyzed by a distinct enzyme. Moreover, we will usually have *K* (the number of proteins available in the proteomics experiment) larger than the number of non-zero elements in **c** (reactions catalyzed by these enzymes), although it is possible that in some situations the number of reactions in the objective function is greater than the number of enzymes, for example when the same enzyme (or combination of enzymes) catalyzes several reactions in the model.

As is clear from the objective function Eq (1), each weight *c*_*k*_ multiplies the corresponding metabolic flux *v*_*k*_. As an example, suppose we identify three proteins 1, 2 and 3 with levels *p*_1_ = 1.0, *p*_2_ = 2.0 and *p*_3_ = 3.0 in a given sample. The corresponding normalized vector is obtained using Eq (4) and is equal to $\tilde{\text{p}}=[1/3;2/3;1]$. For the sake of this example, we assume that these three proteins catalyze three reactions R023, R042 and R128 in the GSMN model. We assume further that protein 1 catalyzes reaction *v*_23_, protein 2 catalyzes reaction *v*_42_ and an AND combination of proteins 1 and 3 catalyze reaction *v*_128_. The objective function to be maximized by FBA is then defined to be:

$$\begin{array}{c} f\left(v\right)=\frac{1}{3}v_{23}+\frac{2}{3}v_{42}+\frac{1}{3}v_{128} \end{array}$$ (6)

where the vector of weights is given by ***c*** = [1/3;2/3;1/3], since the AND expression is calculated using the minimum of the two values in $\tilde{\text{p}}$.

We note that by using data from different time points it is possible to define different objective functions for different time points, something that it is not possible when either the more common objective biomass yield or any other time-independent objective function is used. In this work we assume that our proteomics data for the control group should not be significantly different even for different time points, thus we use an average value of these data to perform FBA for the control condition. Our proposed methodology focus on defining an objective function by using proteomics data. Therefore, our FBA constraints are unaltered and are the same as the ones used with the regular FBA procedure. This way, when we present results of comparisons between our method and the E-flux method, we are comparing two variants of the plain FBA procedure modified by the introduction of proteomics data: one method (E-flux) uses proteomics data to redefine flux constraints and a biomass objective function, while the method proposed here uses proteomics data to define the objective function and unconstrained internal fluxes (as in the regular FBA method).

In this study we have performed comparisons of the proposed methodology with the alternative method E-flux [2]. This method was originally devised to use transcriptomics data to adjust FBA constraints using a “pipe capacity” analogy. Since the E-flux method can also be used with proteomics data, we run FBA using our proposed methodology and the E-flux method with the same set of data and compare the results obtained. We first simulated both methods and the regular FBA procedure using only biomass maximization without any experimental data. Biomass was constrained to the same value as the regular FBA procedure for comparison. We analyzed the results comparing the number of reactions in the model that carried zero flux in the optimal vector *and* are catalyzed by essential enzymes needed for growth as presented by [7]. Here we restrict our analysis to the control experimental condition, where we assume that the objective of the organism is optimal growth, represented by maximization of biomass production. It is expected that in a situation where the organism is not under any biological stress we would end up with a small number of these *essential reactions* (i.e. metabolic reactions catalyzed by essential enzymes).

As a validation step, we performed comparison of prediction error between our proposed method and the E-flux method for a control condition and treatment conditions in different time points. We perform FBA for both methods using randomly chosen subsets of our proteomics data and the remaining subset of proteins to evaluate the prediction of the model in terms of enzyme abundances. This cross-validation step was performed as follows: For one specific time point and experimental condition the set of proteomics data was randomly split in two groups using a 80/20 rule, i.e. 80% of the proteins were used to defined the objective function in our proposed method and to define normalized constraints for the E-flux method, while 20% of the proteins were used as a validation set to compare fluxes and enzyme abundances. This procedure was repeated 16 times with different random splits. The enzymes in the validation set were evaluated in the boolean expressions and the resulting values were used for comparison with the corresponding fluxes obtained with FBA. Normalized squared prediction error was used to evaluate prediction accuracy of the two methods. In doing so, we normalized the error by the number of reactions in the validation set, as without normalization different splits would result in a different number of reactions depending on the boolean expressions evaluated. The validation procedure was repeated and results obtained in different realizations are in agreement. In order to obtain some insight into the metabolic behavior for alternative optima, we perform a final step of flux variability analysis, again comparing both methods. Our results are presented and discussed in the next section.

## Results and Discussion

Using the reconstructed *in silico* genome-scale metabolic network of the *Mycobacterium tuberculosis* presented in [16], we perfomed FBA using three different objective functions, namely (1) biomass yield, (2) proteomics data in the objective function and (3) proteomics data defining the constraints for the corresponding reactions. All these were performed for the control data in order to evaluate the results of the proposed objective function when compared to the results obtained using the two other approaches. The objective function defined with proteomics data was constructed according to the method detailed in the previous section. To use proteomics data to determine constraints for reactions we employed the same method as in the E-flux method for transcriptomic data [2]. For each of the three problems, one run of FBA was performed for each time point (6 hrs, day 2 and day 4). Since the biomass objective is the same regardless of the time point, this was performed only once. This is one advantage of using proteomics data to define the metabolic goal of the cell, since it is clear that metabolic pathway usage profile and goal may change with time. Proteomics gives a useful picture of the metabolic possibilities that the cell may use at each time point.

### Proposed Methodology and E-flux

First we compare the results of FBA for the biomass objective function and the objective function defined using quantitative proteomics data from the control condition only. We must check whether using a proteomics objective function instead of the more common biomass goal, still produces sound flux results. One possible way is to observe the number of reactions carrying zero flux that are considered essential, and where by an *essential reaction* we mean a metabolic reaction catalyzed by enzymes considered essential for growth according to Sassetti et al. [7]. In the following we term these zero-flux reactions catalyzed by essential enzymes (ZFER) and use the number of these reactions as a first metric of quality of FBA results. We assume that reactions catalyzed by enzymes essential for growth should present flux value different from zero. In Table 2 we present the number of ZFERs after performing three FBA simulations in a control condition (where biomass yield, i.e. optimal growth, is assumed in the absence of any biological stress): biomass objective function, proteomics objective function, E-flux method with proteomics constraints. We note that the flux configuration using standard FBA without any proteomics data produces 100 ZFERs. Using our proposed objective reduces the number of ZFERs to 66, likely improving FBA results, since we have a significantly smaller number of fluxes which are catalyzed by essential enzymes that are equal to zero. Using proteomics data to determine flux constraints, as in [2], the number of ZFERs increases to 103. Therefore, we note that the proposed methodology of using proteomics data to define an objective function even in a control condition still produces good results, and with less reactions catalyzed by essential enzymes carrying zero flux. In Table 2 we also present the percentage of essential reactions in the model, since this is different from the number of essential enzymes, because of the combined action of enzymes defined by the boolean expressions explained in the previous section. Although the proportion is very significative, it is interesting to note that in general this issue is not often addressed in articles that study metabolism with FBA (e.g. [1]). The resulting smaller number of reactions with zero flux when utilizing the proteomics objective function may be indicative of extra information brought into the optimization problem by the proteomics data. We note that of all the reactions still remaining with zero flux with the new objective function, most were also observed with the biomass objective, showing that the proteomics objective does not induce different reactions to zero, but only lowers the number of these when compared to biomass maximization, therefore adding knowledge to the problem.

**Table 2:** Number of reactions catalyzed by essential enzymes according to [7] carrying zero flux.

	FBA	PmxObj	E-flux
% ZFERs	54%	46%	57%
enzymes	100	66	103

This table shows the percentage of GSMN-TB reactions catalyzed by essential enzymes that carry zero flux in the optimal vector in the control condition. Number of corresponding enzymes catalyzing these reactions are presented in the second row. Three methods were simulated: FBA with biomass objective function, proposed method with objective function defined by proteomics data (PmxObj) and E-flux method with constraints adjusted with proteomics data.

It is important to discuss further the need for a biomass constraint. In the GSMN, the biomass reaction is a convenience equation defined as a weighted linear combination of macromolecule precursors such as DNA, RNA, protein, other small molecules, essential cell wall components, etc. The biomass reaction used in our simulations with the GSMN-TB *in silico* model is the following:

$$\begin{array}{ccc} & & \;0.214\;\text{PROTEIN}\\ & + & \;0.036\;\text{RNA}\\ & + & \;0.022\;\text{DNA}\\ & + & \;0.050\;\text{SMALLMOLECULES}\\ & + & \;0.006\;\text{PE}\\ & + & \;0.016\;\text{TAGbio}\\ & + & \;0.040\;\text{PIMs}\\ & + & \;0.186\;\text{LAM}\\ & + & \;0.208\;\text{MAPC}\\ & + & \;0.035\;\text{P-L-GLX}\\ & + & \;0.007\;\text{CL}\\ & + & \;0.054\;\text{LM}\\ & + & \;47\;\text{ATP}\\ & = & \;1.0\;\text{BIOMASS}+47\;\text{ADP}+47\;\text{PI} \end{array}$$

where the numerical weights represent the stoichiometric coefficients for each metabolite. We note that mols of several components are needed to obtain one mol of biomass along with 47 ADP and orthophosphate molecules resulting from the hydrolysis of the ATP molecules. The calculations for determination of this biomass reaction are not the focus of the present work and may be found in detail in [16]. It is based on manual curation of literature and reflects the knowledge about the actual composition of cells of *Mycobacterium tuberculosis*. The names of metabolites in this stoichiometric equation follow the nomenclature of convenience used in the GSMN model and can be found in the additional data file 6 from [16]. Here, **PE** (L-1-phosphatidyl-ethanolamine), **TAGbio** (triacylglycerol), **PIMs** (phosphatidylinositol mannosides), **LAM** (lipoarabinomannan), **MAPC** (mycolic-acid-arabinogalactan-peptidoglycan complex), **P-L-GLX** (poly-L-glutamate-glutamine), **CL** (cardiolipin), **LM** (lipomannan), **PI** (phosphate).

Since the biomass function above is an external convenience transport reaction in the model, when we modify the objective function from biomass to the one defined in terms of the proteomics data (which affect mainly intracellular reactions which are precursors of the components in the biomass equation), the consumption and production of biomass components will no longer be maximized, and the resulting optimal flux vector will be different from the one obtained when we assume biomass maximization. Moreover, since the proteomics objective function may produce a metabolic state with biomass flux zero even under the control, it is important to constrain the biomass equation to a minimum value so that conclusions are not off-target. In the above comparison we constrained biomass yield to the same value obtained with regular FBA when maximizing a biomass objective.

It is important to note though, that our goal is not to use proteomics-defined objective functions as a replacement for biomass maximization, but rather for situations of drug stress. As a source of validation however, it is reasonable to expect that, by using control data to define the objective function, the metabolic functioning of the cell should be similar. That is why we performed comparisons for the control condition as well as for the treatment condition.

**Fig 1:**
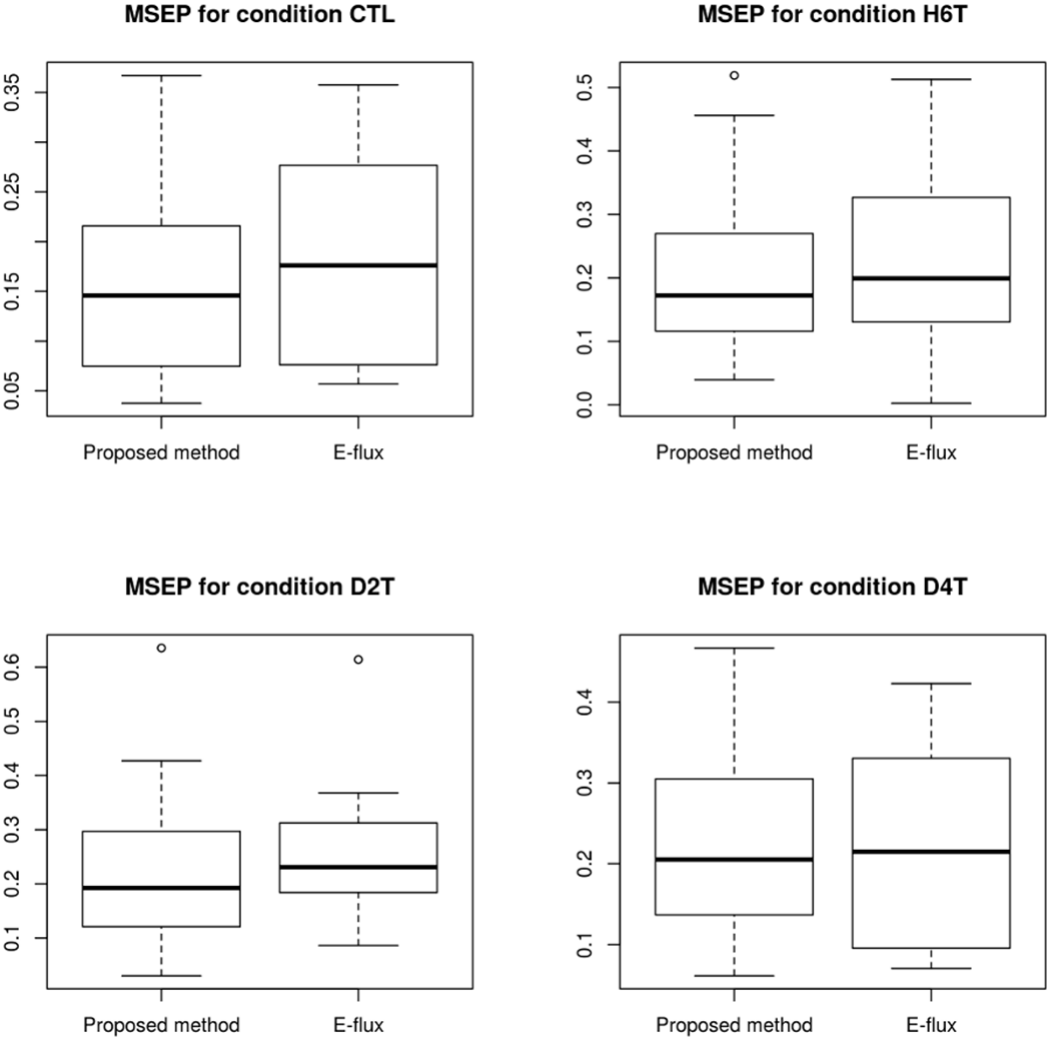
Mean squared error of prediction for the proposed method and the E-flux method for three experimental conditions. The use of proteomics data to define objective functions in FBA yields lower predicion errors.

After having compared essential reactions, we compared our proposed methodology with the E-flux method in terms of prediction error. To this end we performed several runs of FBA with a subset of the proteomics data and validated the results with the remaining subset, as explained in the previous section. Results for mean squared error of prediction are presented in Table 3. These are the mean results of 16 runs of the simulation. In this table we observe that the mean squared error of prediction for the validation group of proteins is lower for the proposed method in all time points. We performed a *t*-test on the difference of the errors for the two methods and report in the last row the *p*-values for a significance level of 95% and 15 degrees of freedom. We observe that even for higher *p*-values, the error difference is consistently lower in the case of using the proposed methodology for all conditions. In Fig 1 we present a boxplot of the errors obtained in each of the 16 folds of the validation procedure for both methods. We observe that in some situations (specific splits of the proteomics set) it is possible to have a lower error for the E-flux method, but in the mean the proposed methodology is consistently better.

**Table 3:** Comparison of the MSEP for the proposed method and the E-flux method.

	CTL	H6T	D2T	D4T
MSEP PmxObj	0.20	0.26	0.21	0.23
MSEP E-flux	0.24	0.34	0.24	0.26
p-value	0.07	0.02	0.13	0.20

This table shows the MSEP (mean square error of prediction) for the proposed method (PmxObj) and the E-flux method with proteomics data for different experimental conditions. The proposed methodology yields lower prediction error in all conditions. (t-test for 95% significance level, 15 degrees of freedom). **CTL** (control), **H6T, D2T, D4T** (treatment condition after 6 hours, 2 days and 4 days. Last row shows p-values of the t-test for the significance of the error differences.

In constraint-based optimization it may be possible that optimal solutions are not unique. Alternative optima represent situations where the metabolic system reaches the same optimal objective function value using a different set of reactions, which is possible due to redundancies inherent to the metabolic network. Therefore, we next use flux variability analysis (FVA) [23] with our proposed method and E-flux to analyze the impact of possible alternative optima. In this paper we choose to evaluate the presence of alternative optimal solutions using the technique of FVA that has been successfully used to obtain insights on alternative optima and is also readily available for simulation with SurreyFBA. Another possibility is the use of the MILP method to enumerate all the possible non-unique optimal solutions. This approach however, in the case of genome-scale metabolic networks, may be computationally intractable due to the exponentially growing number of extreme points that may exist [23, 24]. FVA basically determines the range of variability for each flux in the network due to the presence of alternative optima, so it allows us to study some important features of the behavior of the system due to its redundant network.

Alternative optima are responsible for the situation where different uses of the underlying network of metabolic reactions correspond to the same cellular function [24]. By defining the optimization problem as the maximization of a subset of internal reaction fluxes instead of biomass, it may be expected that the variability in fluxes would be reduced. Since the biomass function is itself a linear combination of several internal fluxes, it could be the case that more redundancy is present in its maximization in contrast to maximizing a set of internal fluxes themselves. This is what is observed with our FVA simulations. In Table 4 we show that by using proteomics to define the objective function indeed results in a lower number of reaction fluxes with high flux variability. For this table we consider reactions with high flux variability those for which the flux range (i.e. maximum flux value minus minimum flux value produced by FVA) is larger than 0.05. This result is interesting since it shows that by using an objective that maximizes specific fluxes according to observed proteomics, redundancy in the network plays a less prominent role than with biomass maximization. We see that with our proposed objective, the goal of the cell is more focused, since a significantly smaller number of reactions allow large magnitude variability in their fluxes in all experimental conditions studied.

**Table 4:** Number of reactions with large flux variability for the proposed proteomics objective function and the E-flux method.

Condition	E-flux	PmxObj
6 hrs Control (replicate 1)	164	72
6 hrs Control (replicate 2)	185	79
6 hrs Mefloq. (replicate 1)	276	72
6 hrs Mefloq. (replicate 2)	248	65
Day 2 Control (replicate 1)	149	72
Day 2 Control (replicate 2)	272	70
Day 2 Mefloq. (replicate 1)	266	70
Day 2 Mefloq. (replicate 2)	267	72
Day 4 Control (replicate 1)	259	72
Day 4 Control (replicate 2)	266	72
Day 4 Mefloq. (replicate 1)	276	68
Day 4 Mefloq. (replicate 2)	298	70

This table shows the number of reaction fluxes with large variability due to alternative optima for the proposed method (PmxObj) and the E-flux method for different experimental conditions. The proposed methodology allows high flux variability to a significantly lower number of reactions in comparison to E-flux in all conditions. The number of reactions for regular FBA (not shown) is similar to the numbers for E-flux. These represent reaction fluxes for which the range (maximum flux minus minimum flux) due to alternative optima is larger than 0.05.

We also observed, for each experimental condition, the flux variability in reactions catalyzed by essential enzymes according to the essentiality criterion in [7]. It is interesting to observe that with the proteomics objective function most of the essential reactions present zero variability. We have verified a variability reduction in all FVA simulations when using the proposed objective function defined by proteomics data. This is a good indication that the incorporation of protein data in the objective function, instead of only using it to determine constraints, represents a promising approach to help discern biologically relevant metabolic flux configurations with the aid of experimental information. In general, we observe that the number of alternative optima is reduced using our proposed objective.

**Fig 2:**
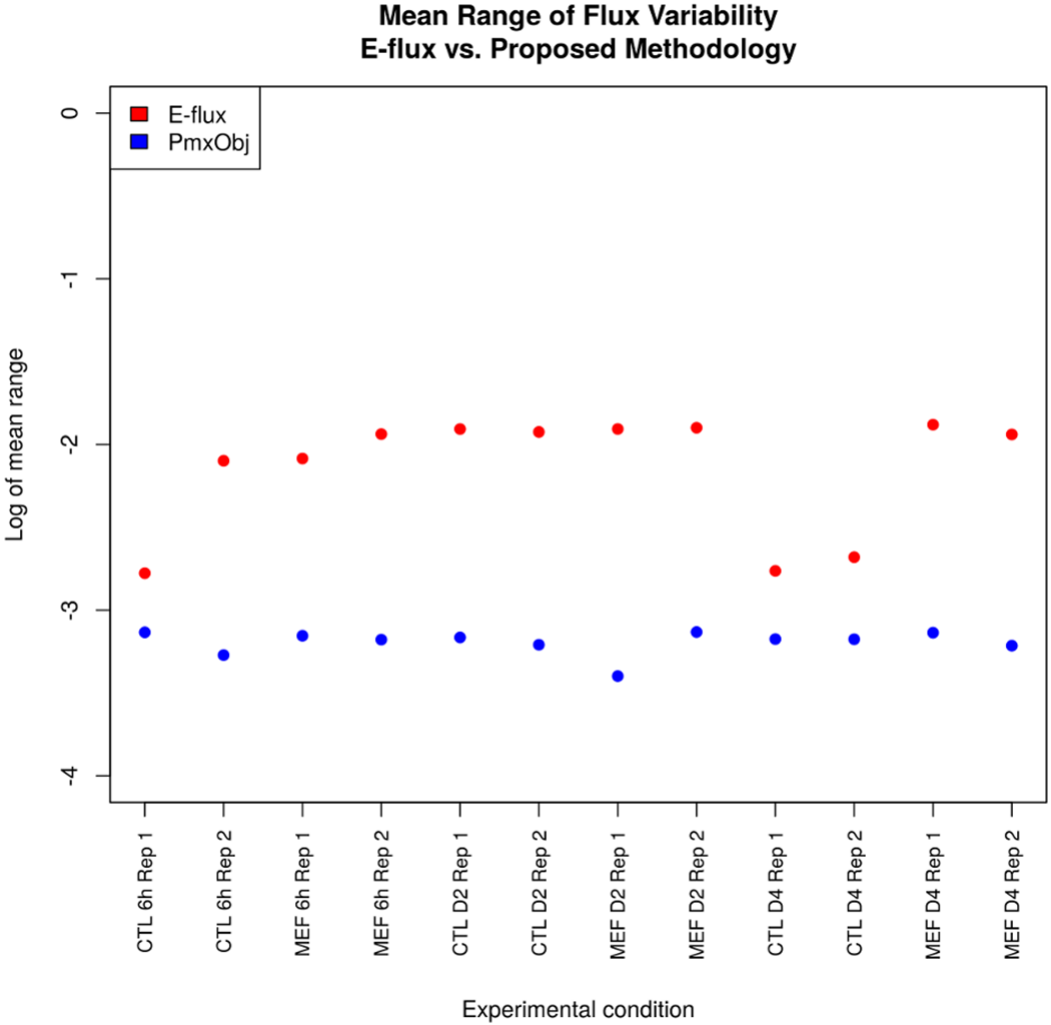
Comparison of flux variability between the proposed method and the E-flux method for all experimental conditions (individual replicates). The use of proteomics data to define objective functions in FBA yields lower mean flux variability due to alternative optimal solutions.

The FVA simulations reveal that, besides reducing the number of reactions that show large variability, using the proteomics objective function also reduces the magnitudes of these variabilities when compared to using the biomass function. Fig 2 shows the logarithm of the mean values of the range of variability in all experimental conditions for our proposed methodology and the E-flux method. From these results we see that the proposed objective function indeed helps in reducing the magnitude of variability, therefore producing an optimal flux vector that is less affected by the presence of possible alternative optima. In all experimental conditions the FVA ranges for the E-flux method are larger than with our proposed methodology.

The obtained results indicate that there are clear advantages to use objective functions defined by experimental proteomics data, mainly in situations of cellular stress (e.g. antibiotics exposure), where biomass maximization may not be the best assumed objective. We believe that more research in this direction is required to further evaluate this new and promising approach to FBA.

### Metabolic Fluxes and Pathways

The metabolic model comprises 856 reactions for which an optimal flux is obtained with FBA. When we modify the objective function from biomass to one defined by proteomics data, out of 856 reactions, a small number (10%) of the reactions in the control condition present significant change in their optimal flux value (i.e. 76 reactions). It is important to note that by using an objective function defined in terms of proteomics data, as mentioned above, implicitly assumes that metabolic fluxes are mainly regulated by enzyme concentrations, disregarding the fact that their activity may also be regulated by metabolite concentrations or other signalling proteins. Nonetheless, it is a useful option whenever none of these extra data is used.

The intracellular fluxes that most greatly varied from one objective function to the other are part of metabolic pathways related to carbon source (glucose) degradation and energy production, as can be seen in the plot for the region with lower indices (in the GSMN-TB model most reactions belonging to pathways of energy transduction such as glycolysis, citrate cycle, energy metabolism have low indexes). Other two reactions with fluxes that varied significantly are R134, R363, due to the presence of high levels of protein *katG*—*Rv1908c* (P9WIE5), for peroxidase-catalase activity, in the control condition. All reaction names and formulae are available in [16] in the additional data file 4.

Many proteins present both enzymatic as well as signal transduction roles in the cell, but in FBA only the enzymes are taken into account. It is therefore possible, that some proteins having high levels in the proteome for roles other than enzymatic activity, will produce spurious results in FBA. One important factor that may cause difficulties when using proteomics data to define the objective function is that enzyme activity is not simply a function of enzyme levels, but is also regulated by metabolite concentrations and other regulation mechanisms. This is actually a fact that can help with the understanding of metabolic regulation. If a proteomics-defined objective function is not close to the one obtained when biomass is maximized, it is possible that the main differences are largely due to lack of information on metabolite concentrations and enzyme regulation. As an example, the high levels of *katG* observed in proteomics may not translate into high enzymatic activity due to the presence of inhibitor molecules or other regulator proteins that are not being accounted for in the simple FBA optimization.

The incorporation of transcription factors [25] and regulatory information onto FBA is not new [26] and it has been shown to improve the results [4]. It is therefore possible that by incorporating regulatory information we may eliminate some of the sources of noise corrupting the FBA predictions. A straightforward approach to add regulatory constraints onto FBA has been presented, for example, in [27]. The method uses knowledge of the transcriptional network of the organism with a set of simple boolean expressions that enables the analysis of metabolic fluxes further constrained by gene regulation.

Lastly, two other facts may also cause interference in the FBA results. First, the limited number of proteins that a proteomics experiment can identify compared to the complete proteome of a cell. Further, the *in silico* metabolic model itself (GSMN-TB) is not complete and many blanks are left in those parts of the metabolic network that are either not known, not implemented in the model, are presently not associated with specific enzymes or even have annotation inconsistencies. These factors, together with the lack of signalling information, may account for the major differences observed when proteomics is used to define an objective function.

After having compared the metabolic flux results in the control condition, we now turn to compare the changes in metabolic configuration using only objective functions defined in terms of proteomics data for both control and drug conditions. We observe the resulting fold-change differences in metabolic fluxes between the two conditions. We limit the comparison to fluxes presenting fold-change larger than 2 or smaller than 0.5. We discuss the significance of the obtained fluxes by comparison with biological knowledge on the behavior of the bacterium when exposed to mefloquine, as presented in [6].

**Fig 3:**
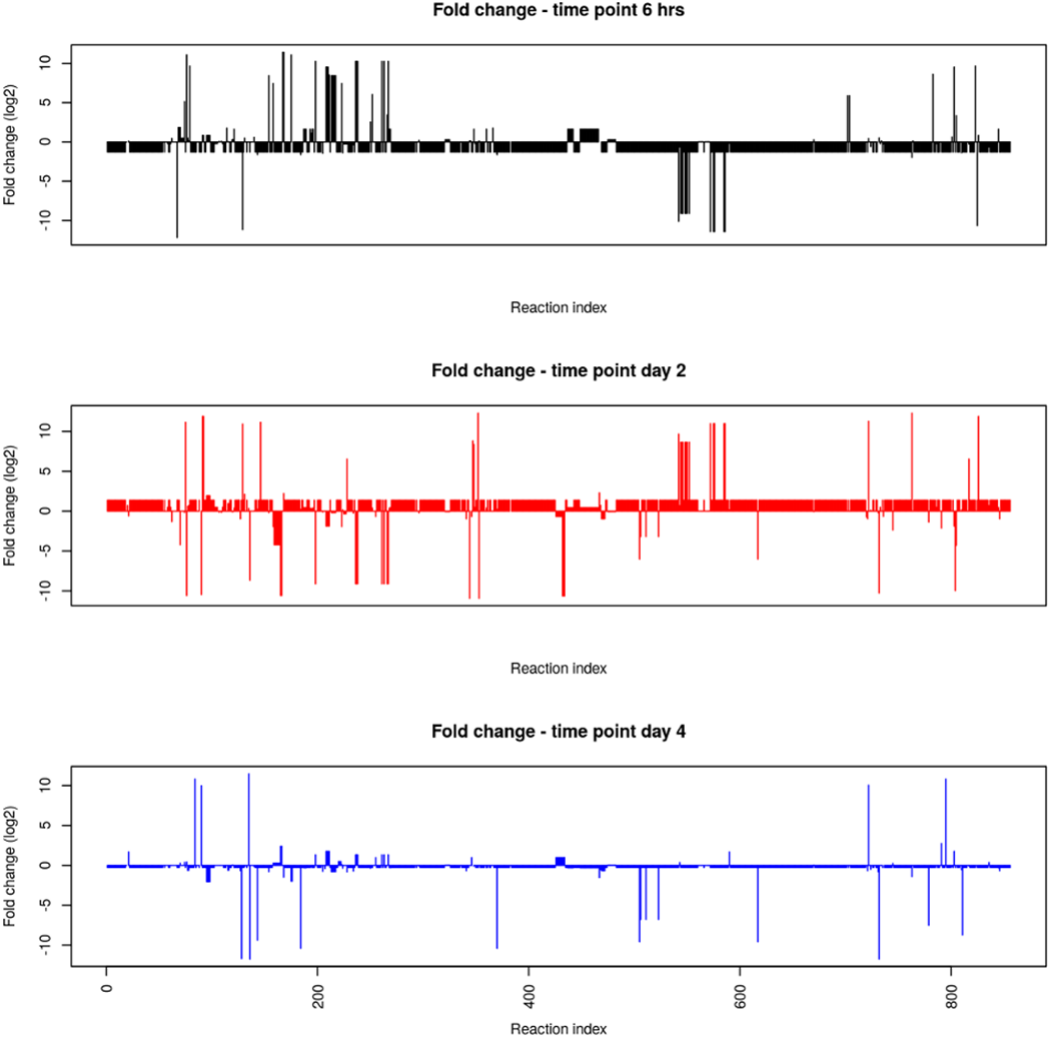
Comparison of fold-change. Logarithm of fold change of metabolic flux for mefloquine to control condition.

The fold-change in flux is presented for each time point in Fig 3. A large number of reactions in the model are not active (i.e. have zero flux) in either condition, so these were set to a fold-change value of one.

According to Table II in [6], upon exposure to mefloquine genes *Rv0904c* (I6WZQ9), *Rv3411c* (I6X784) and *Rv3515c* (I6YCB1) show large fold-change values. None of these three genes are present in our proteomics data. However, the pathways in which these enzymes participate are correctly identified by FBA. For example, large metabolic fold-change is observed in reactions R156, R163 and R495, all pertaining to pathways for lipid biosynthesis, like the FAS1, where *Rv0904c* is the gene encoding the catalyzing enzyme. We note that these are predicted by FBA only on day 2 and day 4, but not on the 6 hours time point. Gene *Rv3411c*, encoding an enzyme participating in the pathway for nucleotide (purine) biosynthesis, although not available in proteomics, catalyzes a reaction in a pathway that is correctly predicted by FBA (in time point 6 hrs) to have larger fluxes upon mefloquine exposure. A similar conclusion can be drawn for the protein encoded by the gene *Rv3515c*. These results indicate that FBA can correctly predict reactions in pathways that have been shown to be more active upon mefloquine exposure.

FBA results also show a large increase in biotin synthesis (R425,R426), which is a precursor molecule in the FAS1 system [21] of lipid biosynthesis and mycolic acid production. This molecule is used in the beginning of the pathway, and it is just sensible that the fluxes of its production would be predicted to be high in earlier time points. Another pathway with several reactions showing increased fold-change are reactions for cell wall synthesis, namely peptidoglycan biosynthesis (R712-R714). It is interesting to note that these reactions are not present in the first time point of 6 hrs, but only in time points day 2 and day 4.

By using a proteomics-defined objective function for FBA allowed us to obtain a metabolic flux configuration on a per-time point basis, something that is not possible with maximization of biomass or any other function that is not time-dependent. We have observed this advantage with the biotin reactions discussed above. As another example, we observe that reaction R003, in the pathway for glycerol metabolism, presents flux changes over time. In the 6 hour sample this flux has fold-change 0.2, changing to 0.5 in day 2 and back to 0.2 in day 4. It is interesting to note that the genes coding for the enzymes that catalyze this reaction, *Rv2249c* (I6X3P8) and *Rv3302c* (I6Y352) are not in the proteomics data, so that the flux is driven indirectly by the expression of other enzymes in the model.

Another reaction with dynamic behavior is observed for reaction R069 in pyruvate metabolism. The enzyme catalyzing this reaction is encoded by *Rv1127c* (O06579), which is not in the proteomics data, however flux through it increases over 100 fold from time point 6hr to time point day 2.

In terms of metabolic pathways, pathways that consistently present large fold-change are degradation of branched-chain amino acids such as valine and isoleucine, of which several reactions are present in the top reactions in all three time points. In the case of studying metabolism to identify metabolic pathways used for survival one should observe the later time points, when bacteria are closer to death. In these time points, large fold-change are observed in pathways for propanoate metabolism (reactions R080), threonine biosynthesis (reactions R227, R231, R891), biotin biosynthesis (reactions R425, R427, R925), peptidoglycan (cell wall) biosynthesis (R712, R714).

We have included a list of metabolic reactions in the *in silico* model that presented the largest fold-change values from the control condition to the mefloquine condition in time points 6 hours, day 2 and day 4, respectively as S1 Table, S2 Table and S3 Table. Each file contains the reactions obtained at each time point. These files can be generated automatically using the available R code, also available as S1 File.

## Conclusion

In this article we evaluated the possibility of using proteomics data to determine an experimental condition-dependent objective function for flux balance analysis. Three main advantages of using proteomics data to define the objective function for FBA are the following:

1. Defining an objective function based on proteomics leads to estimations of flux values that translate directly into variations in metabolism caused by variations in protein content. This can be important for analyzing differential phenotypes, as required for comparing a control condition to a drug condition.
2. Analysis of the metabolism at different time points is facilitated by relying on proteome quantification, which is very closely related to metabolic fluxes in prokaryotic organisms (apart from post-translational modifications and metabolite modulations). The proposed approach allows the derivation of a specific objective function for each treatment condition. Then, an optimal steady-state metabolic phenotype can be obtained by performing FBA for each specific time point.
3. The proposition of using proteomics in the definition of objective functions for FBA can be combined with other strategies. For instance, the proposed objective function can be complemented with a biomass term weighted by a suitable coefficient *c*_*n*+1_. This weighting could be used, for example, to limit the biomass contribution if biomass maximization is not expected to be the main metabolic goal of the cell under a specific experimental condition. Biomass yield can also be constrained directly using minimum and/or maximum reaction constraints in the linear program.

Comparisons were performed in terms of number of reactions and enzymes essential for growth (as published by Sassetti et al. [7], under the control condition) and in terms of prediction error with the E-flux method, an alternative method that uses proteomics data to adjust constraints in FBA. We observed that using an objective function defined in terms of proteomics data produces flux configurations with a lower number of reactions catalyzed by essential reactions carrying zero flux, and consistently produced lower prediction errors compared to the E-flux methodology. With the technique of flux variability analysis (FVA) we observed that with the proposed objective function we reduce flux variability and the impact of alternative optima on the optimal flux solution. This is an important result, since it is desirable that the incorporation of experimental data helps reduce uncertainty in the identification of relevant metabolic distributions in different experimental conditions. The results of FVA simulations show that for the proposed objective function we obtain less reactions with high variability as well as variability with reduced overall magnitudes. Finally, using the proposed approach, it was possible to identify pathways with increased metabolic activity after mefloquine exposure that had already been identified previously [6], providing support for more studies using proteomics data to define metabolic objectives in FBA.

Although we have performed flux balance analysis without using signaling information, its incorporation may help diminish the difficulties associated with proteins that perform both enzymatic as well as regulatory/signaling roles inside the cell environment. This possibility may be considered in future work.

## Supporting Information

S1 Dataset Zip file containing proteomics experimental data in Scaffold format. (ZIP)

S1 File Zip file containing R code for the proposed method, and metabolic model in CSV and SBML formats. (ZIP)

S1 Table Metabolic reactions with largest flux fold-change for time point 6 hours. (CSV)

S2 Table Metabolic reactions with largest flux fold-change for time point day 2. (CSV)

S3 Table Metabolic reactions with largest flux fold-change for time point day 4. (CSV)
